# Evaluation of different enrichment methods for pathogenic *Yersinia* species detection by real time PCR

**DOI:** 10.1186/s12917-014-0192-9

**Published:** 2014-08-29

**Authors:** Maialen Arrausi-Subiza, Jose Carlos Ibabe, Raquel Atxaerandio, Ramon A Juste, Marta Barral

**Affiliations:** 1 Department of Animal Health, Basque Institute for Agricultural Research and Development (NEIKER), Berreaga 1, Derio-Bizkaia, 48160, Spain

**Keywords:** Yersinia enterocolitica, Yersinia pseudotuberculosis, PBSMSB, Wild boars, Real time PCR, Enrichment

## Abstract

**Background:**

*Yersiniosis* is a zoonotic disease reported worldwide. Culture and PCR based protocols are the most common used methods for detection of pathogenic *Yersinia* species in animal samples. PCR sensitivity could be increased by an initial enrichment step. This step is particularly useful in surveillance programs, where PCR is applied to samples from asymptomatic animals. The aim of this study was to evaluate the improvement in pathogenic *Yersinia* species detection using a suitable enrichment method prior to the real time PCR (rtPCR). Nine different enrichment protocols were evaluated including six different broth mediums (CASO, ITC, PSB, PBS, PBSMSB and PBSSSB).

**Results:**

The analysis of variance showed significant differences in *Yersinia* detection by rtPCR according to the enrichment protocol used. These differences were higher for *Y. pseudotuberculosis* than for *Y. enterocolitica*. In general, samples incubated at lower temperatures yielded the highest detection rates. The best results were obtained with PBSMSB and PBS2. Application of PBSMSB protocol to free-ranging wild board samples improved the detection of Y. enterocolitica by 21.2% when compared with direct rtPCR. *Y. pseudotuberculosis* detection was improved by 10.6% when results obtained by direct rtPCR and by PBSMSB enrichment before rtPCR were analyzed in combination.

**Conclusions:**

The data obtained in the present study indicate a difference in *Yersinia* detection by rtPCR related to the enrichment protocol used, being PBSMSB enrichment during 15 days at 4°C and PBS during 7 days at 4°C the most efficient. The use of direct rtPCR in combination with PBSMSB enrichment prior to rtPCR resulted in an improvement in the detection rates of pathogenic *Yersinia* in wild boar and could be useful for application in other animal samples.

## Background

*Yersiniosis* is the third most commonly reported zoonosis in humans in Europe, although the number of reported *Yersiniosis* cases in humans has been decreasing since 2006 [1].

Culture and PCR based protocols are the most common used methods for detection of pathogenic *Yersinia* species in animal samples. Currently, there are official methods to isolate *Yersinia enterocolitica* and *Yersinia pseudotuberculosis* from foods, water and environmental samples [2]-[4]. Nonetheless, culture methods need to improve their sensitivity and specificity to obtain more information about the disease, especially when wild animal samples are studied. It is difficult to achieve a multivalent isolation method suitable for all *Yersinia* or only for pathogenic *Y. enterocolitica* and *Y. pseudotuberculosis*[5]-[7]. So it is not rare that some infected animals are missed by isolation methods. Regarding PCR, one of the most relevant problems for *Yersinia* detection is that sensitivity decreases in samples with low *Yersinia* concentration and high bacterial background. In these cases, an enrichment step improves the detection of the agent by PCR [8]-[10]. This step is particularly useful in surveillance programs, where PCR is applied to samples from asymptomatic animals.

Some authors have used a selective enrichment step before PCR including Tryptone Soya (CASO) broth to detect *Y. enterocolitica* from pig tonsils [8],[11] or *Y. enterocolitica* and *Y. pseudotuberculosis* in tonsils and fecal samples from wild boars [12],[13]. Irgasan Ticarcillin Chlorate (ITC) broth has also been used prior to PCR to detect *Y. enterocolitica* from pig fecal samples [14], as well as Peptone sorbitol bile (PSB) broth in the detection of *Y. enterocolitica* from bulk milk and cheese [9] and fecal samples obtained from dairy farms [15].

An enrichment step is also commonly used before *Yersinia* isolation including selective enrichment broths like CASO, ITC and PSB [2],[16],[17]. But also with non-selective enrichments like phosphate-buffered saline broth (PBS), PBS supplemented with 1% sorbitol and 0.15% bile salt (PBSSSB) or PBS supplemented with 1% manitol and 0.15% bile salt (PBSMSB) [18]-[22].

The aim of this study was to evaluate the improvement in pathogenic *Yersinia* species detection by use of a suitable enrichment method prior to the real time PCR.

## Methods

### Sample collection

Ethical approval is not required by a specific committee since animals used in the present study were not sacrificed for research purposes. Samples were collected from legally hunted wild boars in the frame of a wildlife health surveillance program developed in the Basque Country (North of Spain) (Basque Government Project id: VEPIFAUS-61.0292.0). Wild boars had been shot by accredited hunters and tissue samples were taken in the field in collaboration with competent local authorities. Samples were collected in individual containers properly identified and sent to the laboratory where they were stored at -80°C until analyzed.

### Experiment 1: evaluation of different enrichment protocols

#### General procedure

Tonsil samples obtained from four wild boars naturally infected with pathogenic *Yersinia* were used as the reference material. Tonsil samples, collected during the 2010–2011 hunting season, were analyzed by direct rtPCR following the protocols described below. Two of them (wild board 1 and 2) were positive to *Y. enterocolitica* and the other two (wild board 3 and 4) to *Y. enterocolitica* and *Y. pseudotuberculosis* and were selected for the experiment 1 in order to evaluate different enrichment protocols.

**Figure 1:**
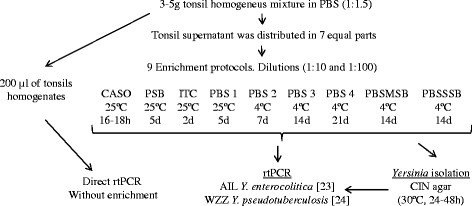
Scheme of the enrichment protocols evaluated in this study.

A tonsil sample (3–5gr) from each wild boar was weighed and aseptically cut into small pieces. These were then mixed with phosphate-buffered saline (PBS) in a 1:1.5 proportion (sample:PBS) and homogenized in an Stomacher (Lab-Blender 80) until a homogeneous mixture was achieved. The supernatant was removed and 200 μl were stored at -20°C for direct *Yersinia* PCR detection. The rest of the supernatant was distributed in 7 aliquots that were used to evaluate 9 different enrichment protocols (Figure 1). The same supernatant aliquot was used for enrichment protocol PBS 2, 3 and 4. The enrichment broths used were CASO (Fluka, Germany), ITC (Fluka, Germany), PSB (Fluka, Germany), PBS [23], PBSSSB [6] and PBSMSB [6]. Two different dilutions, 1:10 and 1:100 were evaluated in each enrichment protocol. Each sample was incubated for the periods and temperatures indicated in Figure 1. Then, 200 μl of each supernatant aliquot was removed for *Yersinia* detection by rtPCR and the rest of the suspension was stored at -20°C for microbiological studies.

#### DNA extraction

200 μl of tonsil homogenate and 200 μl of each enrichment medium supernatant were digested with proteinase K (20 mg/ml) (Invitrogen, Carlsbad, CA) and ATL buffer (Qiagen, Hilden, Germany). DNA extraction was performed with Qiamp®DNA Blood mini kit (Qiagen, Hilden, Germany) following manufacturer instructions with minor modifications (Load wash 1 with 800 μl of Buffer AW1 and load elution with 80 μl of buffer AE). Finally, DNA was measured in NanoDrop ND-1000 spectrophotometer (Thermo Scientific, Inc.).

#### Detection of Y. pseudotuberculosis and pathogenic Y. enterocolitica by rtPCR

Between 150 and 200 ng of DNA from each sample were used for *Yersinia* detection by rtPCR. Pathogenic *Y. enterocolitica* was detected by *ail* gen amplification with R-real 9A (5-CCCAGTAATCCATAAAGGCTAACATAT-3), F-real 10A (5-ATGATAACTGGGGAGTAATAGGTTCG-3) primers and *ail* probe (5-FAM-TGACCAAACTTATTACTGCCATA-TAMRA-3) [24]. PCR cycling parameters included an enzyme activation cycle at 50°C for 2 minutes and an initial denaturation cycle at 95°C for 2 minutes followed by 45 cycles at 95°C for 15 seconds and 60°C for 1 minute. *Y. pseudotuberculosis* was detected by *wzz* gene amplification with Y. pseu F (5-AGAAGAYGGTTTRGATAAAMGAGCGT-3), Y. pseu S (5-AACYGAGGGTGAMAATGAATATCGCT-3), Y. pseu A (5-GGAAAACATCAGCATTAACGATGGTA-3), Y. pseu R (5- GGAAAACATCAGCATTAACGATGG-3) primers and Y. pseu TM probe, (5-FAM-CAACAAGTCACGAGCRTCTGTCGGTGT-TAMRA-3) [25]. PCR cycling parameters included an enzyme activation cycle at 50°C for 2 minutes and an initial denaturation cycle at 95°C for 2 minutes followed by 45 cycles at 95°C for 15 seconds, 60°C for 33 seconds and 72°C for 30 seconds. In the two reactions Express qPCR Supermix Universal Invitrogen™ kit was used.

#### Yersinia isolation

20 μl of supernatant from 1/100 dilutions of each enrichment protocol were inoculated in selective Cefsulodin-Irgasan-Novobiocin (CIN) Agar (BioMérieux, France) and incubated at 30°C for 24–48 h for *Yersinia* isolation. When one or more characteristics red “bull’s-eye” colonies, surrounded with a transparent area of 1 mm appeared, some of those colonies were streaked directly onto Triple Sugar Iron (TSI) Agar (OXOID LTD, England) and incubated at 30°C for 24 h. Tubes without gas production, no hydrogen sulphide formation (no blackening of the medium), glucose fermentation and lactose/sucrose fermentation (butt yellow and yellow slant) for *Y. enterocolitica* or glucose fermentation only (butt yellow and red slant) for *Y. pseudotuberculosis* were selected. Colonies with these TSI characteristics were picked and plated on Blood agar (Agar Columbia; BioMérieux, France) and incubated at 30°C for 24 h. Colonies compatible with *Yersinia* spp. were selected and identified by VITEK system (BioMérieux, France). These colonies were also homogenized in 500 μl of PBS and 50 μl of this mixture were incubated 10 minutes at 100°C in a wet bath and then 10 minutes in ice. Then the mixture was centrifuged 10 minutes at 15600 × *g* and 5 μl of supernatant were used for pathogenic *Yersinia* identification by rtPCR following the detailed procedures.

If no colonies were obtained with an enrichment protocol supernatant, the procedure was repeated with a 1/10 dilution.

Serotyping was performed by slide agglutination using a commercial *Y. enterocolitica* O:1, O:2, O:3, O:5, O8 and O:9 antisera (Denka Seiken, United kingdom), *Y. enterocolitica* O:27 antisera (SIFIN, Berlin, Germany) and *Y. pseudotuberculosis* O:1 to O:6 antisera (Denka Seiken, Tokyo, Japan).

### Experiment 2: procedure validation with wild board samples

Tonsil samples were obtained from 66 free ranging wild boars between 2010 and 2012. These samples were analyzed by rtPCR, directly and after processing with the enrichment protocol selected from experiment 1 evaluation, in order to verify its effectiveness. 150 mg tonsil from each wild boar were disrupted and homogenised with 30 balls of zirconium (1,3 mm Chrome steel beads-Biospec Products-USA) and 750 μl of TE buffer using a ribolyzer (TissueLyzerII-Qiagen-Germany). 200 μl supernatant were submitted to DNA extraction following the steps mentioned in “*DNA extraction*” section. The rest of each tonsil (1–4 g) was aseptically cut and mixed with the enrichment medium selected, according to the conditions described in Figure 1. Then 200 μl of enrichment supernatant was removed for DNA extraction. Then these samples were used to detect *Y. pseudotuberculosis* and pathogenic *Y. enterocolitica* by the rtPCR protocols described before.

### Statistical analysis

Inverse Cycle Threshold values (ICT) obtained in rtPCR from each tonsil homogenate and after each one of the 9 enrichment procedures were analyzed as the quantitative dependent variable for the main experimental categorical effects: protocol, dilution and incubation temperature. The ICT was calculated by subtracting the cycle threshold (Ct) obtained in each sample from the Ct value considered negative that in this study was 46. So that a negative sample ICT would be 0. Statistical analysis was carried out with the SAS 9.3 statistical package (SAS Inc., Cary, NC, USA). After checking overall data consistency (graph plot) and distribution type (Kolmogorov-Smirnov normality test) of ICT with the UNIVARIATE procedure, values were submitted to analysis of variance with the PROC GLM statement for main effects testing. For comparison of means according to these independent variables for each *Yersinia* species p values for groups were tested with the Tukey-Kramer correction for multiple comparisons. Validation of the selected enrichment protocol with naturally infected wild boar samples was performed by McNemar’s test and the simple kappa coefficient of agreement in the TABLES statement of the SAS PROC FREQ. P values less than 0.05 were considered statistically significant.

## Results

### Experiment 1: evaluation of different enrichment protocols

*Y. enterocolitica* and *Y. pseudotuberculosis* rtPCR was carried out on 72 and 36 enrichment supernatants respectively. PCR positive results were obtained in 93.1% for *Y. enterocolitica* and in 97.2% for *Y. pseudotuberculosis*. Not all enrichment protocols identified all the samples as positive. ITC failed to detect *Y. enterocolitica* in one sample (wild boar 2) and *Y. pseudotuberculosis* in wild boar 3. CASO, PBS1, PBS3 failed to detect *Y. enterocolitica* in wild boar 3 and PBSSSB failed to detect *Y. enterocolitica* in wild boar 2. PBS2, PBS4 and PBSMSB protocols gave better or similar Ct results when compared with direct rtPCR. ITC, PBS1 and PSB protocols showed worst performance (Table 1).

**Table 1:** **rtPCR results obtained from the enrichment protocols evaluated in experiment 1 for** ***Y. pseudotuberculosis*** **and** ***Y. enterocolitica***

**Enrichment protocol**	***Y. enterocolitica*** **rtPCR**			***Y. pseudotuberculosis*** **rtPCR**		
	**Pos (Ct ≤)**	**Pos (Ct >)**	**Neg.**	**Pos (Ct ≤)**	**Pos (Ct >)**	**Neg.**
CASO	5	2	1	4	0	0
PSB	3	5	0	0	4	0
ITC	5	2	1	0	3	1
PBS1	5	2	1	0	4	0
PBS2	7	1	0	4	0	0
PBS3	6	1	1	4	0	0
PBS4	5	3	0	4	0	0
PBSMSB	5	3	0	4	0	0
PBSSSB	6	1	1	3	1	0

Pos (Ct ≤): rtPCR positive result after enrichment with Ct value minor or similar (±0.5) than Ct values from direct rtPCR.

Pos (Ct >): rtPCR positive result after enrichment with Ct value major than Ct values from direct rtPCR.

Neg: rtPCR negative result.

**Figure 2:**
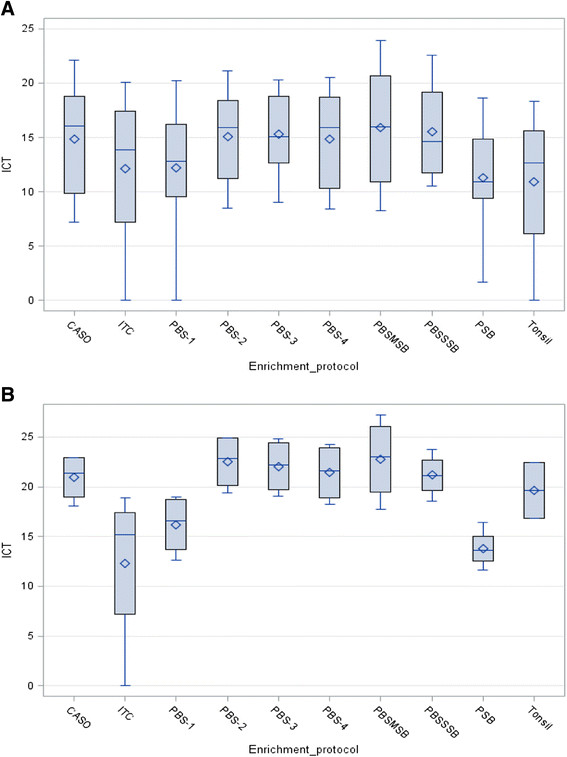
**Distribution of ICT values obtained from the evaluated enrichment protocols shown as box-plot graphs.** Each of the 10 box-plot represents summary statistics of the ICT values for a particular enrichment protocol and tonsil homogeneous mixture (Tonsil) for *Y. enterocolitica***(A)** and *Y. pseudotuberculosis***(B)**. Boxes represented the 25% and 75% percentiles, the horizontal lines inside boxes indicate the ICT median values, the diamond shape represent the ICT mean and the vertical lines extended to the ICT minimum to maximum values. The number of samples studied in each enrichment protocol was 8 for *Y. enterocolitica* and 4 for *Y. pseudotuberculosis*. Attending to tonsil homogeneous mixture they were 4 for *Y. enterocolitica* and 2 for *Y. pseudotuberculosis*.

The analysis of variance showed significant differences in *Yersinia* detection by rtPCR according to enrichment protocol used (p = 0.0141). Those differences were higher for *Y. pseudotuberculosis* than for *Y. enterocolitica* (p < 0.0001) (Figure 2). Attending to the incubation temperatures, samples incubated at lower temperatures showed better ICT values (p < 0.0001) than samples incubated at higher temperatures. The higher ICT values were obtained with PBS2, PBS3, PBSMSB and PBSSSB protocols (Table 2), being PBSMSB the protocol with the best ICT values for both *Yersinia* species although no significant differenced were observed (p = 0.8065). No significant differences were observed in relation with the sample dilutions tested, although the best ICT values were obtained with 1/10 dilution (data not shown).

**Table 2:** **ICT mean and standard deviation values obtained from each enrichment protocol for** ***Y. enterocolitica*** **and** ***Y. pseudotuberculosis***

**Enrichment protocol**	***Y. enterocolitica***		***Y. pseudotuberculosis***	
	**ICT Mean**	**ICT SD**	**ICT Mean**	**ICT SD**
CASO	14.85	5.50	20.96	2.40
ITC	12.14	7.05	12.31	8.42
PBS 1	12.17	6.32	16.21	3.06
PBS 2	15.08	4.50	22.52	2.79
PBS 3	15.30	3.98	22.07	2.77
PBS 4	14.85	4.73	21.43	2.98
PBSMSB	15.92	5.71	22.76	4.16
PBSSSB	15.51	4.49	21.17	2.15
PSB	11.33	5.32	13.80	1.98
Tonsil	10.90	7.75	19.65	3.95

SD: ICT standard deviation.

Tonsil: Direct PCR in tonsil homogeneous mixture.

*Y. pseudotuberculosis* was isolated from two of the enrichment supernatants, PBS4 (1/100) from wild boar 4 and PBSSSB (1/100) from the wild boar 3, and confirmed by rtPCR. No agglutination was detected by serotyping with the antisera used. No other pathogenic *Yersinia* were isolated from the remaining of 1/100 dilution supernatants, or 1/10 dilutions, but in some cases non-pathogenic *Yersinia* were isolated.

Attempts were made with alkali treatment (0.5% KOH) [2] of the supernatant before CIN agar inoculation without any new isolation. Direct plating of the 4 tonsils allowed the isolation of *Y. pseudotuberculosis* from wild boar 4.

### Experiment 2: procedure validation with wild board samples

According to experiment 1 results, PBSMSB enrichment during 14 days at 4°C in a 1/10 dilution was selected to verify its effectiveness. 18 of the 66 (27.3%) free ranging wild boars were positive to *Y. enterocolitica* using PBSMSB before rtPCR while only 6.1% (4/66) of the samples were positive by direct rtPCR. PBSMSB application improved the detection of *Y. enterocolitica* by 21.2% (p = 0.001) (Table 3). In relation with *Y. pseudotuberculosis* no significant differences were observed when direct rtPCR results were compared with PBSMSB before rtPCR, detecting 10.6% (7/66) and 13.6% (9/66) positives respectively (Table 4). The use of both protocols in combination increased by up to 10.6% the number of *Y. pseudotuberculosis* positive samples (p < 0.05), that is, detecting 14 positive wild board.

**Table 3:** ***Y. enterocolitica*** **rtPCR results obtained from free ranging wild board tonsils by direct rtPCR and with PBSMSB enrichment prior to rtPCR**

	**PBSMSB enrichment + rtPCR**		
**Direct rtPCR**	**Positive**	**Negative**	**TOTAL**
**Positive**	2	2	4
**Negative**	16	46	62
**TOTAL**	18	48	66

**Table 4:** ***Y. pseudotuberculosis*** **PCR results obtained from free ranging wild board tonsils by direct rtPCR in relation with PBSMSB enrichment prior to rtPCR**

	**PBSMSB enrichment + rtPCR**		
**Direct rtPCR**	**Positive**	**Negative**	**TOTAL**
**Positive**	2	5	7
**Negative**	7	52	59
**TOTAL**	9	57	66

## Discussion

There are many studies that evaluate the effectiveness of different enrichment protocols for pathogenic *Yersinia* isolation, including selective or general medium broths at diverse incubation temperatures during variable periods of time [10],[23],[26]-[29]. However, to the best of our knowledge, this is the first time that this evaluation is made prior to PCR, as the majority of studies use a single enrichment step, being CASO and ITC the most commonly used [5],[8],[11]-[14].

In spite of using a limited number of samples with the same animal origin, our results showed that enrichment protocol choice could have a real impact in the detection of *Y. pseudotuberculosis* and *Y. enterocolitica* by PCR that could result in misdiagnosis when applied to field samples.

Enrichment protocols where incubation was carried out at low temperatures were more sensitive and reliable when compared to others with incubation at 25°C. This can be explained by *Yersinia* ability to multiply at refrigeration temperatures. In many other studies that compare different enrichment protocols, cold enrichment has been reported to be more efficient to isolate both *Yersinia* species from tonsils samples [17],[30],[31]. But also from another samples like intestinal content [17],[29] or tongue [32]. Some authors reported that cold enrichment increased the multiplication and isolation of non-pathogenic *Yersinia* that could difficult the isolation of the pathogenic ones [20],[29]. It seems not to be a problem when specific PCR to detect pathogenic *Yersinia* is used after the enrichment protocol.

PBSMSB was the protocol that showed the best ICT result for both *Yersinia* species, followed by PBSSSB and PBS3 for *Y. enterocolitica* and by PBS2 and PBS3 for *Y. pseudotuberculosis*, although differences were no significant, but PBS3 and PBSSSB failed to detect *Y. enterocolitica* in one sample each one. Many authors use PBS based cold enrichment protocols before *Yersinia* isolation obtaining satisfactory results [19]-[22], although no references were found in relation with their use before PCR. Cold enrichment with PBSMSB has been shown to be more effective than ITC for the recovery of *Y. enterocolitica* from pig tonsils, faeces and carcass swabs [18],[28]. Similar results were observed for *Y. pseudotuberculosis* recovery from pig tonsils, intestinal content, faeces or pluck set [17],[29],[31]. However different results were observed when this enrichment was applied to meat samples, as Van Damme reported better isolation rate of *Y. enterocolitica* using PSB at 25°C for 2 days when compared with PBSMSB in cold enrichment [26].

The application of a non-selective enrichment step could be very useful when animal samples are studied, as it can favour detection of the majority of strains of *Y. enterocolitica* and *Y. pseudotuberculosis*.

Regarding sample dilution, no differences were observed when 1/10 or 1/100 dilutions were used, but the use of 1/10 dilution resulted in a more efficient and less expensive method.

Low isolation rates were achieved when all the enrichment supernatants and tonsils were inoculated in CIN agar. This is in agreement which what has been observed in other studies comparing effectiveness of PCR and culture, as culture methods seems to have lower sensitivity [11],[12],[14]. *Y. pseudotuberculosis* was isolated from the two infected animals but in each case from a different enrichment supernatant and directly from the tonsil without any enrichment in one of them. These results do not allow to conclude which would be the better procedure in order to isolate *Y. pseudotuberculosis* from field samples. On the other hand, it was not possible to isolate pathogenic *Y. enterocolitica* from any of the 4 infected wild boards, although additional attempts were made with alkali treatment and direct plating. CIN agar is the most widely used culture medium for *Yersinia* spp. isolation but it has been reported that it can inhibit the growth of some strains of *Y. enterocolitica* and *Y. pseudotuberculosis*[33]. Another possibility is that enrichment supernatant conservation at -20°C without cryoprotectants facilitates *Y. enterocolitica* inactivation, although *Y. pseudotuberculosis* isolation from two of the same samples has been recorded.

More efforts should be made in order to isolate pathogenic *Yersinia* strains from PCR positive animals, especially when samples from wildlife are studied, since these species could act as reservoirs of many different strains [16]. In those cases more than one parallel or sequential isolation step would be required for the isolation of pathogenic *Yersinia*[29] and the use of cryoprotectants in case of enrichment supernatant freezing. The use of more than one medium for both enrichment and plating will result in higher recovery rates, as no single selective medium is available for all strains isolation [6].

*Y. pseudotuberculosis* isolated strains did not agglutinate with any of the used antisera that includes the most commonly detected human serotypes. So another serotypes from O:7 to O:14 were probably infecting wild boars in the Basque Country although these serotypes seems to be not commonly found in Europe [6].

The application of PBSMSB protocol to the 66 free ranging wild boar samples clearly increased the detection of *Y. enterocolitica* when compared with direct rtPCR. Nevertheless the number of *Y. pseudotuberculosis* positive samples detected directly or before PBSMSB enrichment was similar, although only two samples were positive with both procedures. Some samples were positive after enrichment but negative by direct PCR (16 for *Y. enterocolitica* and 7 for *Y. pseudotuberculosis*). Since in asymptomatic carriers the concentration of pathogenic *Yersinia* is usually low, PCR detection can be difficult. In such cases, an enrichment step should allow the multiplication of the bacteria up to reach a concentration that can be detected by rtPCR. On the other hand, there were seven samples (2 for *Y. enterocolitica* and 5 for *Y. pseudotuberculosis*) that were negative after enrichment but positive by direct PCR. It could be postulated that they were dead bacteria that were lost the enrichment step implied its dilution and consequently PCR was not able to detect them [34]. Other possible explanation is that sample conservation at -20°C during, in some cases, a long time, could inactivate the *Yersinia*. It would be recommendable the use of both protocols, direct rtPCR and PBSMSB enrichment step before rtPCR, when surveillance programs to detect pathogenic *Y. enterocolitica* and *Y. pseudotuberculosis* are carried out, especially if reservoir species are included.

## Conclusions

The data obtained in the present study indicate a difference in *Yersinia* detection by rtPCR related to the enrichment protocol used, being enrichment with PBSMSB during 15 days at 4°C the most efficient one. Nevertheless, the use of direct PCR in combination with PBSMSB enrichment prior to rtPCR results in an improvement in the detection rates of pathogenic *Yersinia* in wild boar and could be useful for application in other animal samples. However, more efforts should be made to improve the isolation of pathogenic *Yersinia*, especially *Y. enterocolitica*.

## Competing interests

The authors declare that they have no competing interests.

## Authors’ contributions

MA and JCI performed the laboratorial analyses. RJ, MA and MB carried out the statistical analysis. MA and MB wrote the manuscript and RA and RJ contribute to draft it. MA and MB conceived and designed the experiments and MB coordinated and supervised the study. All authors participated in results interpretation and read and approved the final manuscript.
